# Moebius strips of chiral block copolymers

**DOI:** 10.1038/s41467-019-11991-3

**Published:** 2019-09-09

**Authors:** Zhen Geng, Bijin Xiong, Liquan Wang, Ke Wang, Min Ren, Lianbin Zhang, Jintao Zhu, Zhenzhong Yang

**Affiliations:** 1 Key Lab of Material Chemistry for Energy Conversion and Storage of Ministry of Education, School of Chemistry and Chemical Engineering, Huazhong University of Science and Technology, Wuhan, 430074 China; 2 Shanghai Key Lab of Advanced Polymeric Materials, School of Materials Science and Engineering, East China University of Science and Technology, Shanghai, 200237 China; 3 Institute of Polymer Science and Engineering, Department of Chemical Engineering, Tsinghua University, Beijing, 100084 China

**Keywords:** Materials chemistry, Supramolecular chemistry, Soft materials

## Abstract

The Moebius topology (twisted, single-sided strip) is intriguing because of its structural elegance and distinct properties. Here we report the generation of block copolymer Moebius strips via a fast self-assembly of chiral block copolymer polystyrene-*block*-poly(D-lactide acid) (PS-*b*-PDLA) in tetrahydrofuran/water mixed solvents. The Moebius strip is formed by morphological evolution from large compound micelle (LCM) to spindle-like micelle (SLM) and then to toroid with a 180° twist along the ring. Mechanism insight reveals that a subtle balance of crystallization of PDLA and microphase separation between PS and PDLA chains dominates the formation of Moebius strips. An intriguing helix-helix transition occurs during the chiral transfer from microphase to assemblies, which is driven by relaxation of the internal stress within SLM related to orientated stretching of PS chains. Mesoporous chiral channels can be generated within Moebius strips after removal of PDLA, which are interesting in chiral recognition, separation and asymmetric catalysis.

## Abstract (web summary)

The Moebius topology is intriguing because of its structural elegance and distinct properties. Here the authors report block copolymer Moebius strips via a fast self-assembly of chiral block copolymer polystyrene-*block*-poly(D-lactide acid) in tetrahydrofuran/water mixed solvents.

## Introduction

Moebius strip with a fascinating topological toroidal structure, consists of a surface with just one side^1–3^. In daily life, pulley belts are usually in the form of Moebius strip to wear the surface equally. The non-oriented surface endows Moebius strips with unique performances in electric circuit resonators^4^, wire-loaded copper cavity^5^, and metamaterials^6^. Conjugated molecules with a Moebius topology have gained significant concerns due to the stunning structure and unique aromaticity^7^. Artificially, a Moebius strip can be formed by sequentially snipping a paper ring, adding a twist and then joining the two ends together^8^. Yet, spontaneous formation of this peculiar geometric nanostructure still remains challenging. Tanda et al. prepared a crystalline NbSe_3_ Moebius strip using a spherical droplet as a spool^9^. The Moebius strip topology is mainly resulted from Se surface tension thus bending of the NbSe_3_ crystal ribbon. Bending the ribbon into a toroidal structure and further twisting along the ring are critical to form the Moebius strip.

Block copolymers (BCPs) can self-assemble into a wide variety of supramolecular nanostructures with adjustable sizes and morphologies depending mainly on the composition, molecular weight, and molecular architecture^10–13^. Interestingly, toroidal structures are of particular interest and are efficiently achieved by end-to-end closure of rod-shaped assemblies or by perforation of a spherical/disc-like micelle^14–19^. Jiang and coworkers reported that BCPs toroids could either form through the closure process of rodlike micelles or through perforation of vesicles, and both the formation mechanism and ring size were critically dependent on shear force^14^. Recently, Lin et al. discovered a THF- and temperature induced end-to-end closure of rodlike micelles of poly(_γ_-benzyl-L-glutamate)-*graft*-poly(-ethylene glycol) (PBLG-*g*-PEG) copolymers into toroidal micelles, and the formation process of PBLG-*g*-PEG toroids was quite similar with cyclization reaction of molecules^17^. The formation of the toroidal structures is instructive to further understand biological processes such as toroidal condensation of DNA^20,21^, aggregation of amyloid peptides^22^. It is noted that the polymer toroidal structures just demonstrate a simple ring-like shape, and no twisted structures are present within the toroids^14,15,23,24^. It is anticipated that a twisting structure can be introduced to the toroids by chiral transfer or symmetry breaking during the self-assembly of chiral BCPs. Many variables including hydrophobic interaction, electrostatic interaction, hydrogen bonding, crystallization, steric effect, external confinement and *π*–*π* stacking can be finely balanced to achieve supramolecular structures with special chiral topology^25–29^. Cheng et al. deeply investigated the hierarchical chiral transfer of synthesized chiral liquid crystalline polyesters during their isothermal crystallization and pointed out that it was the interaction and packing scheme instead of the lower-level chirality itself that determine the next level chirality; meanwhile, the transfer of chirality from one length scale to another is neither automatic nor necessary^30^. However, construction of polymeric Moebius strips with a twisted structure remains unsolved.

Herein, we demonstrate a facile strategy to fabricate BCPs Moebius strips via the self-assembly of semi-crystalline chiral diblock copolymers (BCPs*) in a mixed solvent of tetrahydrofuran (THF) and water. Polystyrene-*block*-poly(D-lactide acid) (PS-*b*-PDLA) and PS-*b*-PLLA are selected as the model chiral BCPs. Addition of water to the BCPs* solution leads to spherical micelles formation and structural evolution to form Moebius strips. Based on kinetically trapped intermediate superstructures along the self-assembly, formation mechanism of Moebius strips is proposed. The twisted structure along the strip ring is originated from the hierarchical chiral transfer of PDLA blocks from chain  conformation to supramolecular structure. After degradation of PDLA, chiral mesoporous channels are generated inside the Moebius strips.

## Results

### Preparation of block copolymer Moebius strips

**Fig. 1:**
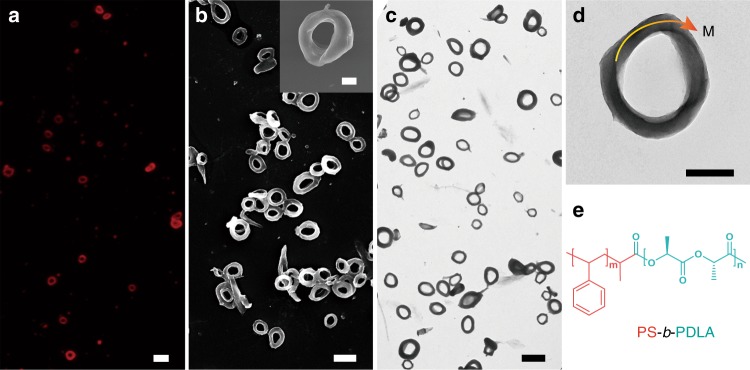
Block copolymer Moebius strips. **a** Fluorescence microscopy (FM) image of fluorescence dye-labeled PS-*b*-PDLA toroidal micelles. Nile red (<0.001 wt%) was added to the BCP solution prior to addition of water. **b** SEM and **c** TEM images of the PS-*b*-PDLA toroidal micelles. Inset in (**b**) and (**d**) are representative magnified SEM and TEM image of the M-twisted PS-*b*-PDLA Moe*b*ius strip. **e** Chemical structure of the PS-*b*-PDLA. Scale bars: 2 μm in (**a**–**c**); 500 nm in (**d**) and inset of (**b**)

Typically, PS_202_-*b*-PDLA_97_ was dissolved in THF, a good solvent for both blocks (the solubility parameters of PS, PDLA, and THF are 18.8 (MPa)^0.5^, 20.2 (MPa)^0.5^, and 18.6 (MPa)^0.5^, respectively^31^). The two blocks become less dissoluble in the mixed solvents when adding water. After fast adding water to the polymer solution under mild stirring, a series of assemblies with various structures are obtained at varied water fraction (see the Methods section for experimental details). In the THF/water (77/23, v/v) mixture, toroidal assemblies form. When the assembly is labeled with Nile red, a red ring contour is discerned under fluorescence microscope (Fig. 1a), confirming the existence of the toroidal micelles in THF/water mixture. The toroidal structure is further confirmed by scanning electron microscopy (SEM), transmission electron microscopy (TEM), and atomic force microscopy (AFM) investigation (Fig. 1b, c, and Supplementary Fig. 1). The average outer diameter (*D*_out_) is measured to be 1.14 ± 0.23 μm, width (*w*) of 0.24 ± 0.06 μm, and average height (*h*) of 0.37 ± 0.09 μm (Supplementary Fig. 2). The discrepancy between the width and height implies that cross section of the ring is not exactly circular. Especially, magnified SEM and TEM images reveal that part of toroids possess 180° twisted topology along the ring, which is the feature structure of so-called Moebius strip (inset in Fig. 1b and Fig. 1d). The TEM tomography 3D image further evidences the twisted structure at a fixed direction along the ring (Supplementary Movies 1 and 2). Two successive cycled traces can be plotted along with the wider face on the ring without crossing the edge, also implying that the Moebius strips possess a 180° twist (Supplementary Fig. 3a). Side view of Moebius strip from STEM and SEM image further reveals a figure-of-eight shaped structure (Supplementary Fig. 3b and 3c), which is the structural characteristics of the Moebius strip^9^. Notably, it is well-known that Moebius strips have two different types according to the handedness of twist along the ring, i.e., left-handed (M-twisted) and right-handed (P-twisted). Therefore, we investigated the ring structure of PS-*b*-PDLA toroids in detail by calculating the ratios of M-twisted Moebius strips, P-twisted Moebius strips, and achiral toroids from both SEM and TEM images (Supplementary Fig. 4). The statistical results indicate that nearly half of the PS-*b*-PDLA toroids possess Moebius strips topology and the handedness of the twist along the ring of Moebius strips is majorly left-handed. Similarly, the toroidal assemblies of PS-*b*-PLLA can also be prepared (Supplementary Fig. 5a). Even though the handedness of twist along the ring of PS-*b*-PLLA toroids is hard to be detected due to their irregular toroidal morphology which may be caused by the molecular composition of PS-*b*-PLLA (see the below discussion), P-twisted PS-*b*-PLLA Moebius strips could commonly be observed (Supplementary Fig. 5b–e).

### Formation kinetics for the toroidal assemblies

**Fig. 2:**
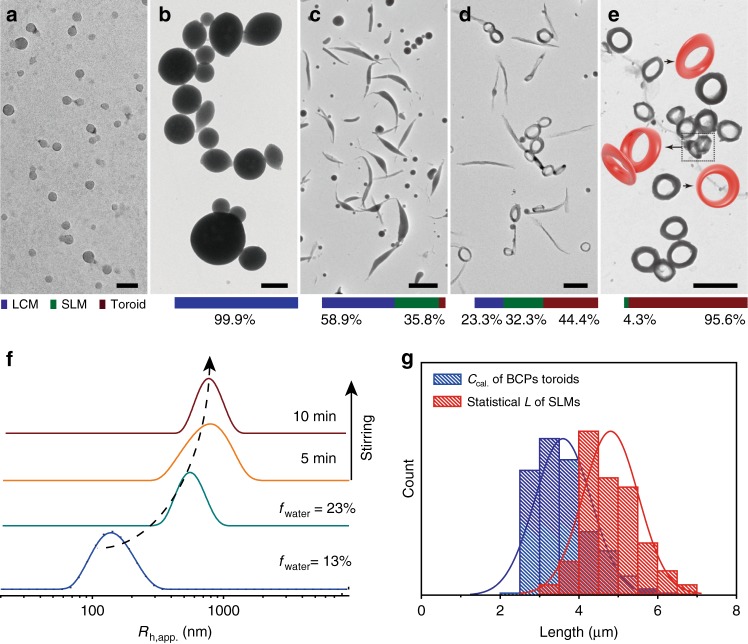
Morphological evolution during the self-assembly of PS-*b*-PDLA into toroidal micelles. TEM images of the intermediate assemblies obtained at varied water content (volume fraction, *f*_water_): **a** 13%, **b** 18%, **c** 23%. **d**, **e** Assemblies achieved after 5 and 10 min upon adding water, respectively. Frequency bar charts of the assembled morphologies (LCMs, SLMs, and toroids) at given water content and varied time duration, statistic more than 300 particles. **f** *R*_h,app._ distributions of the assemblies in solution depending on time duration. **g** Length of the spindles (*L*) (Fig. 2c) and calculated perimeter (*C*_cal._) of the Moebius strips. Scale bars: 100 nm in (**a**); 500 nm in (**b**); 2 μm in (**c**–**e**)

Moebius strip is featured with both toroidal and twisted structures. Intermediate structures were recorded along the self-assembly by fast adding water (1.0% (v/v) per second) into the micelles suspension. At early stage, small spherical micelles (SSMs) of tens nanometers appear at 13.0% of water content (Fig. 2a). Self-assembly of PS-*b*-PDLA is thermodynamic equilibrium owing to a sufficient chain relaxation. Continuous adding water to 18.0% will drive the SSMs to coalesce into large compound micelles (LCMs) with an average diameter of 0.63 ± 0.18 μm (Fig. 2b and Supplementary Fig. 6). The coalescence is very fast within 10 s. Further adding water to 23%, the LCMs transformed to spindle-like micelles (SLMs) rather than fused with each other into larger ones (Fig. 2c). This morphological transition is correlated with semi-crystallization of the PDLA chains, which will be discussed in detail later. Eventually, the SLMs are spontaneously transformed into the toroidal micelles within 10 min (Fig. 2e). Notably, This process is similar to the formation of PBLG-*g*-PEG toroids reported by Lin et al.^17^. In their study, curvature and connection of end-to-end are responsible for forming the toroids, which is driven by the arrangement of side chains on the PBLG backbone. No twisted structure is presented along the toroidal ring of PBLG-*g*-PEG. Obviously, our case is quite different. Formation of our twisted toroids occurs at higher hierarchical level by the spontaneous looping of SLMs, which is driven by the resistance acting oppositely to the movement direction. Shearing flow facilitates bending of SLMs thus forming the rings (Supplementary Fig. 7)^14,16^. Besides, the chemical composition for the surface of intermediate assemblies is revealed to be varied from mostly PDLA chains for SSMs to both of PDLA and PS for LCMs, SLMs, and toroids (Supplementary Fig. 8 and Supplementary Table 1), indicating that the solubility of PDLA chains become comparable with PS along with increased water content in the mixed solvents. Therefore, the hydrophobic solvation of PDLA chains is proposed to be the driving force for the fast coalescence of SSMs into LCMs. Notably, the storage time has negligible effects on the morphology of the Moebius strips (Supplementary Fig. 9), implying the strips are rather stable. Distribution frequency of LCMs, SLMs, and toroidal micelles along the morphological evolution at given water contents is estimated. While fraction of the spheres decreases, fraction of the toroids increases along the transformation of LCMs to SLMs and then to toroids. The apparent hydrodynamic radius (*R*_h,app._) of the BCPs* intermediate assemblies changes (Fig. 2f), consistent with the TEM results. The length of spindles (*L*) (Fig. 2c) is slightly longer than the calculated perimeter (*C*_cal._) of the toroids (*L*/*C*_cal._ ≈ 1.34), implying that the toroidal structure is formed by the end-to-end closure of single spindle and the two ends of SLM are partially overlapped at the junction (Supplementary Fig. 10).

### Crystallization induced deformation of LCMs into SLMs

**Fig. 3:**
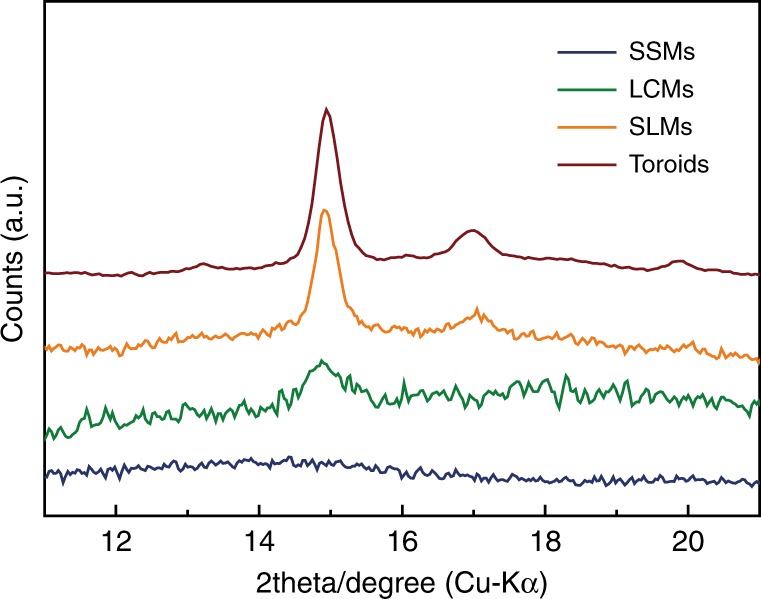
Crystallinity of the intermediate assemblies. WAXS spectra for SSMs, LCMs, SLMs, and PS-*b*-PDLA toroids

Non-spherical morphologies are usually enthalpy unfavorable due to inhomogeneous interfacial energy around the external surface^32^. It is hypothesized that the interesting morphological transition from LCMs to SLMs might be entropy driven by inadequate chain relaxation during the fast self-assembly. As a comparison, another self-assembly of PS-*b*-PDLA was performed by slowly adding water (0.2% (v/v) per min). Lozenge lamellar crystals were achieved rather than the toroids (Supplementary Fig. 11a)^31^. This is related with the sufficient relaxation of polymer chains to induce the crystallization of PDLA. In other words, chain relaxation becomes restricted when fast adding water. The microphase separation and crystallization during the self-assembly are both determined by the rate of adding water. Notably, the tail of SLMs displays a lozenge structure (Supplementary Fig. 11b), quite similar to the lamellar crystal achieved by slow water addition. Therefore, the spontaneous morphological transformation from LCM to SLM and also the final thickness of SLM should be associated with the competition among interfacial interaction between surface of assemblies and mixed solvents, stretching of PS chains, and crystallization of PDLA blocks, which could induce directional deformation of LCMs^33,34^. Wide angle X-ray scattering (WAXS) of the intermediate assemblies during formation of the toroidal micelles was performed to characterize crystallinity of the PDLA phase (Fig. 3). No obvious diffraction peaks are present in the SSMs, while a typical characteristic crystalline diffraction pattern of β form (2*θ* = 14.8° and 17.3° corresponding to the diffraction of (200) and (131) lattice plane of β form crystal in PDLA) for semi-crystalline PDLA exists in both the SLMs and Moebius strips. The spontaneous morphological transformation from LCMs to SLMs is indeed caused by the semi-crystallization of PDLA. SLMs and toroids display the same diffraction patterns. This further confirms that the toroidal BCP micelles are formed by closure of the spindles.

### Formation mechanism of toroidal assemblies

**Fig. 4:**
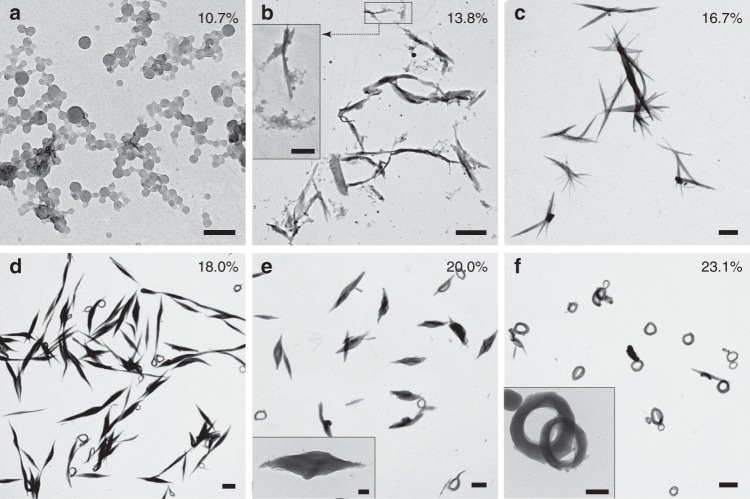
Effect of water content on the self-assembly of PS-*b*-PDLA. TEM images of representative structures formed in THF/water mixtures with varied water content (*f*_water_): **a** 10.7%, **b** 13.8%, **c** 16.7%, **d** 18.0%, **e** 20.0%, and **f** 23.1%. The insets in (**b**) and (**e**) are higher-magnified images of structures formed and inset in (**f**) is a Rused ring-like assembly observed very occasional on the grid. Scale bars: 200 nm in (**a**); 2 μm in (**b**–**f**); 500 nm in insets

Crystallization of BCPs in solution is mainly dominated by solvent quality^35^. To further explore the intriguing morphological transitions of PS-*b*-PDLA assemblies from SSMs to LCMs, and then to SLMs, we performed analogous experiments in which the fast water addition rate was fixed while the water content was varied. After allowing the solutions to stand for 1 day, samples were placed on grids and observed by TEM (Fig. 4). In contrast to the kinetically trapped intermediate assemblies during the formation of toroids as shown in Fig. 2, the assemblies observed under different water content are mostly like thermodynamic structures. At low water content (*f*_water_ = 10.7%, Fig. 4a), SSMs formed. Unexpectedly, further addition of water induced the formation of SLMs among a broad water content range (13.8–20.0%, Fig. 4b–e), while the preformed structure of LCMs was absent. This result indicated that the previously observed LCMs were transient state during the formation of SLMs. Notably, the SLMs can also form through aggregation and fusion of SSMs when the water content is relatively lower (13.8%, Inset in Fig. 4b). These two kinetically dependent formation mechanisms of SLMs implied that the PDLA chains are preferred to crystallize under such solvents condition. The short-axis diameter of SLMs increased along with the addition of water which is slightly resemble with lozenge lamellar crystals obtained during slow water addition condition, implying that the hydrophobic interaction between PDLA chains and solvents will promote the crystallization of PDLA chains. Moebius strips can be observed occasionally among the SLMs at a somewhat higher water content (>18.0%) and finally become the dominant morphology when the water content reaches 23.1%. The increase of water content not only enhance the hydrophobic interactions between polymer chains and mixed solvents, but also strengthen the interfacial tensions which will cause the stretching of PS chains and thus hinder the development for crystallization of PDLA chains. Therefore, the toroidal morphologies are most likely resulting from the combination of crystallization and microphase separation involved with chiral transfer of semi-crystalline PS-*b*-PDLA. Interestingly, a Rused ring-like assembly was found very occasionally on the grid, which further proven the supramolecular cyclization reaction for SLMs into toroidal micelles. Both of the crystallization and chiral transfer of BCPs* strongly depended on the molecular composition of the copolymer^31,36^. Thus, the self-assembly of PS-*b*-PDLA with different PDLA content was also investigated (Supplementary Fig. 12). Compared with PS-*b*-PDLA to form Moebius strips (volume fraction of PDLA, *f*_PDLA_, is calculated to 0.35), PS-*b*-PDLA with shorter PDLA (*f*_PDLA_ = 0.28) self-assembled into large compound micelles (LCMs), whereas PS-*b*-PDLA with longer PDLA (*f*_PDLA_ = 0.40) self-assembled into mixture of SLMs and toroidal micelles. These results indicate that formation of PS-*b*-PDLA toroids is the consequence of subtle balance between crystallization and chiral transfer involved microphase separation. Block length of PDLA chains could affect both the crystallization of PDLA and microphase separation between PS and PDLA, further dominating the self-assembled structures. Moreover, we tried a two-step assembly process of PS-*b*-PDLA (Supplementary Fig. 13) and find that the self-assembly of PS-*b*-PDLA into toroidal micelles is pathway-dependent, which implies that the generated toroidal micelles are thermodynamic metastable assemblies.

### Origination of the twist along the ring

**Fig. 5:**
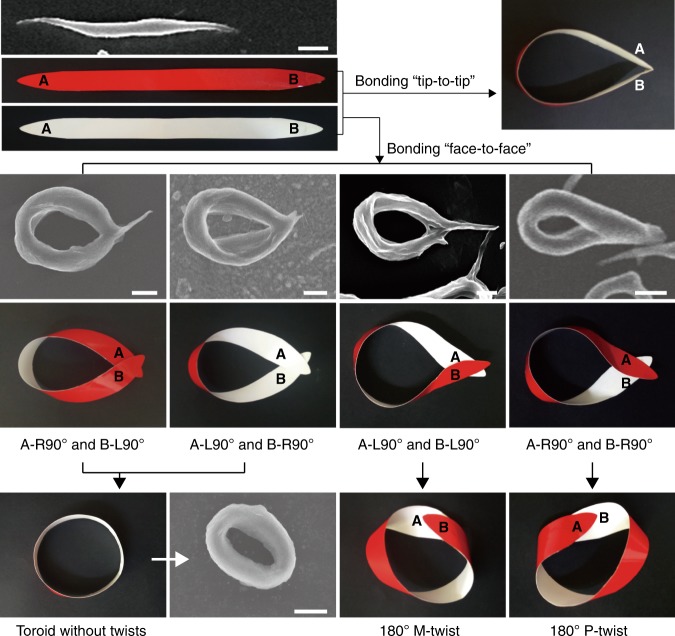
Mechanism insights in the formation of BCPs Moebius strips. Schematic representation of the probable modes for the closing of SLMs into toroidal micelles: the two ends (A and B) of SLMs could bond to each other tip-to-tip or more efficiently through four types of face-to-face way (A-R90°&B-L90°, A-R90°&B-R90°, A-L90°&B-L90°, and A-L90°&B-R90°). Three kinds of toroids (toroid without twists, 180° M-twisted toroid, and 180° P-twisted toroid) could form after closing of SLMs into toroids. Scale bars: 500 nm

The twisted structure along ring is another characteristic for the Moebius strips. We captured the closing procedure of SLMs (Supplementary Fig. 14) and found that the twist expressed supermolecularly along the Moebius strips were most likely generated during this procedure (Fig. 5). During this process, both ends of SLMs (marked as A and B in Fig. 5) preferred to rotate 90° to realize efficient closing, where the bonding area was larger in such face-to-face situation than bonding in tip-to-tip mode. Generally, there are four modes for efficient closing: end A rotates 90° clockwise (Right-handed) and end B rotates 90° anticlockwise (left-handed) (abbreviated as A-R90°&B-L90° in Fig. 5), A-R90°&B-R90°, A-L90°&B-L90°, and A-L90°&B-R90°. If the SLMs were closed through A-R90°&B-L90° or A-L90°&B-R90° mode, no twists along the ring could be formed. Closing through A-R90°&B-R90° mode would induce 180° P-twist along the ring of Moebius strips, whereas closing through A-L90°&B-L90° mode induced 180° M-twist. Notably, the above-mentioned four modes for closing of SLMs would take place equiprobably if no additional influence was introduced. Yet, from the statistical results of the ratios of PS-*b*-PDLA toroidal micelles with different twisted handedness (Supplementary Fig. 4), we could notice that the equiprobability for each mode was broken. And formation possibility of M-twisted Moebius strips was ~4-fold higher than that of P-twisted Moebius strips (40.3% for the former while 8.5% for the latter). This phenomenon is the so-called symmetry breaking^37^, and the driving force for this symmetric breaking would be very interesting.

**Fig. 6:**
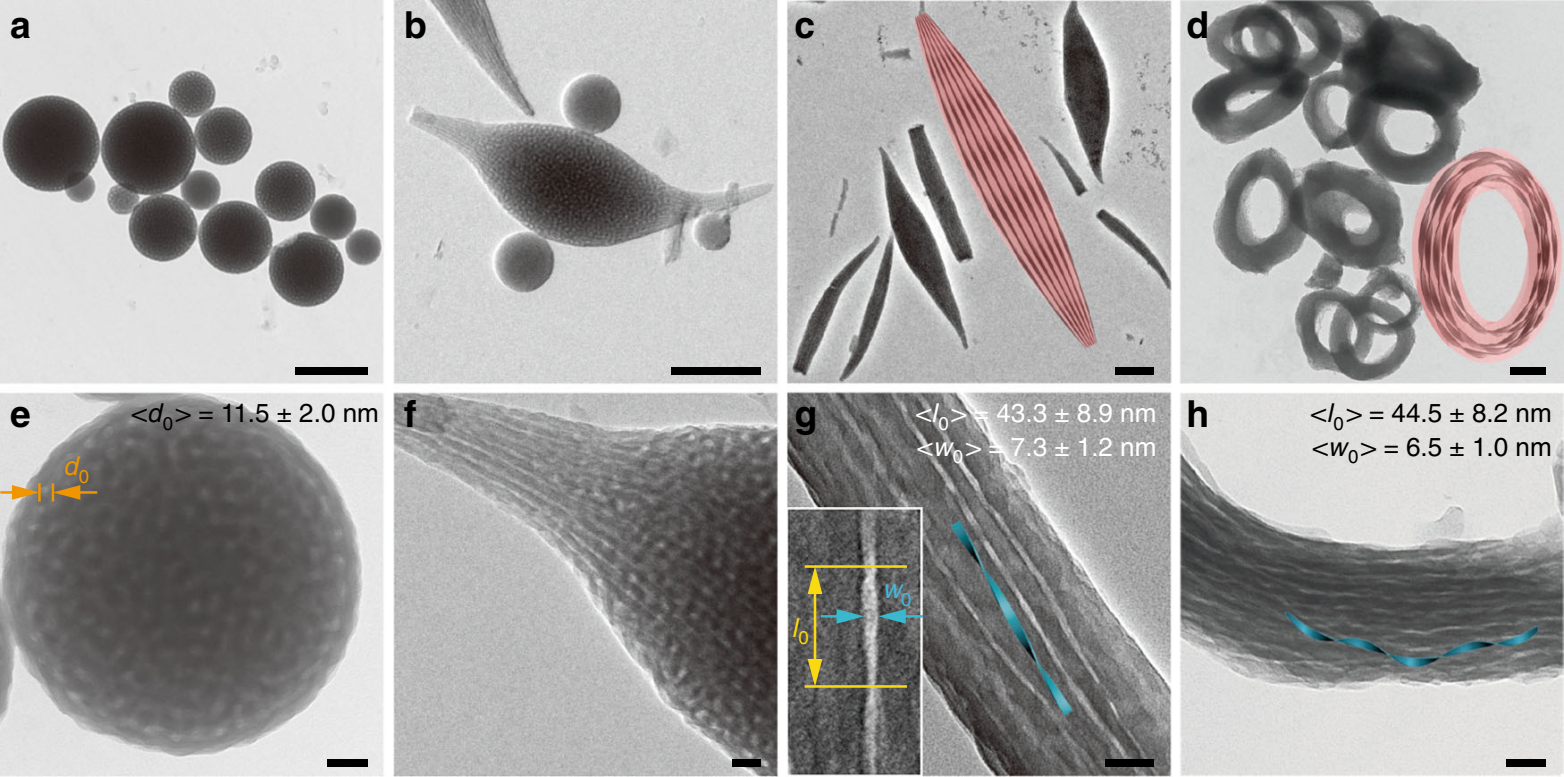
Anisotropic colloids containing mesoporous chiral channels. TEM images of the intermediate assemblies after removal of PDLA microdomains. **a** LCMs. **b** Deformed LCMs. **c** SLMs. **d** Toroidal micelles. **e**–**h** are higher-magnified TEM images in (**a**–**d**), respectively. Schematic description of SLMs and Moebius strips containing roughly parallel mesoporous chiral channel were shown as cartoon images in (**c**) and (**d**), respectively. Scale bars: 500 nm in (**a**–**d**); 50 nm in (**e**–**h**)

A careful electron microscopy analysis of the SLMs provides the information that partial of SLMs possessed M-twisted structure along the long axis (Supplementary Fig. 15). This result implied that the above-mentioned driving force might take place during the structural transformation from LCMs to SLMs. To deeply reveal this structural transformation, the PDLA microdomains within the intermediate assemblies are selectively removed by hydrolysis. A bicontinuous-like internal structure is achieved within the LCMs while the PDLA microphase within the SLMs shows a string of right-handed twisted ribbon geometry (Fig. 6a–c and e–g, respectively). From local features of a deformed LCM which is typical intermediate structure for the transformation from LCM to SLM, the PDLA microphase evolution from bicontinuous-like structure to a quasi-1D twisted ribbon geometry which orientates along the long axis of spindles is further confirmed (Fig. 6b). Moreover, the nanostructure of PDLA microphase within Moebius strips is also revealed to be a string of twisted or helical ribbons (Fig. 6d). It seems that PDLA crystalline structures in rings were relatively more regular than that in SLMs (narrower half-peak width in SAXS, Supplementary Fig. 16). We noticed that the feature sizes of PDLA ribbons (width and pitch length) in spindle and ring were similar. Meanwhile, during the cycling of SLMs into rings, part of PDLA twisted ribbons transformed into helical ribbons (as schematically shown in the inset of Fig. 6d), the later topological structure was energetically more stable than the former^26^. Thus, after selective removal of the PLA microdomains, anisotropic particles (SLMs or toroids) containing roughly parallel mesoporous chiral channel could be obtained which might be promising in catalysis, separation, and template synthesis of chiral composites. Notably, we speculate that the SLMs with different chiral ends (rotated 90° in a clockwise or counter-clockwise manner) might possess different internal nanostructures despite that no obvious difference for PDLA microphases could be found (Supplementary Fig. 17), which will be discussed in detail below. We also carefully calculated the feature size of PDLA microphase within LCMs and SLMs, and found that diameter of section of PDLA microphase within LCMs (*d*_0_) is 11.5 ± 2.0 nm, and the twisted ribbons have an average periodic pitch distance (*l*_0_) of 43.3 ± 8.9 nm and width (*w*_0_) of 7.3 ± 1.2 nm (thickness of PDLA ribbons is too small for efficient count) (insets in Fig. 6). Obviously, feature size of section for twisted ribbon within SLM is much smaller than that for PDLA microphase within LCM, implying a significant conformation change for PDLA chains during the structural transformation.

**Fig. 7:**
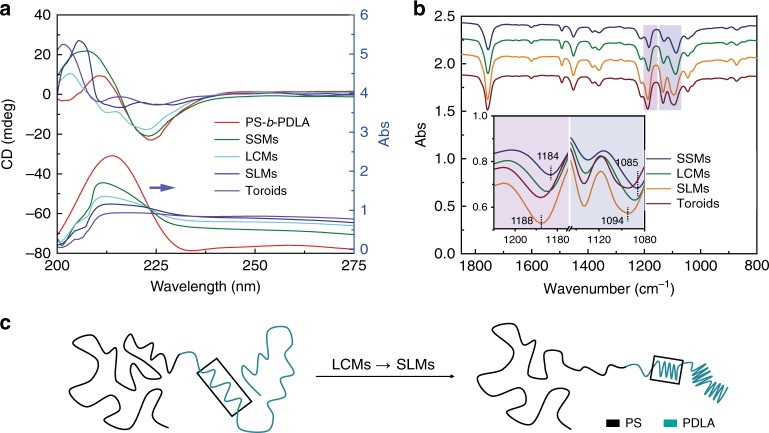
Conformation change of PDLA chains during the self-assembly. **a** CD (top) and UV-vis (bottom) spectra of PS-*b*-PDLA in dilute THF solution, and the assemblies in mixed solvents obtained at varied water content during the formation of BCPs toroidal micelles. **b** FT-IR spectra of the assemblies. **c** Schematic illustration of conformation change for PDLA chains from loosely staggered conformation to compact gauche conformation corresponding to the morphological transformation

The chain conformation variation of PDLA block in the BCP solution during adding water was characterized by circular dichroism (CD) and UV-Vis spectroscopy (Fig. 7a). In dilute THF solution, PLA shows a positive CD signal from PLLA and a negative one from PDLA at 213 nm. The characteristic absorption band is attributed to the n → π* transition of carboxylate chromophore (Supplementary Fig. 18a). Meanwhile, both the PS-*b*-PLLA and PS-*b*-PDLA in dilute THF solutions show an absorption band at 213 nm in UV-vis spectra, similar with enantiomeric PLA. PS-*b*-PDLA displays a CD pattern similar to a standard β-strand structure with a positive band at *λ* ≈ 211 nm and a minimum at *λ* ≈ 224 nm, and the PS-*b*-PLLA displays opposite Cotton effects (Supplementary Fig. 18b)^38^. THF is a good solvent for PDLA chains, and thus PDLA chains are mostly to adapt into loosely staggered conformation. Addition of water causes gradual blue shifts of band both in UV-vis spectra and CD pattern, implying the collapse of PDLA into more compact conformations (Fig. 7a). As mentioned above, WAXS results indicate that PDLA adapts into helical conformation within β form crystal. Moreover, the shifts for FT-IR peaks of the C–O–C vibrations in the range from 1050 to 1220 cm^−1^ for PDLA (from ∼1085 to ∼1094 cm^−1^, from ∼1184 to ∼1188 cm^−1^) further verifies the change of PDLA chains into more compacted conformations (Fig. 7b)^39–41^. Accordingly, a significant conformation change of PDLA chains from loosely staggered conformation to compact gauche conformation (e.g., right helix conformation) occurs corresponding to the morphological transformation from LCMs to SLMs (Fig. 7c).

The above results imply that the PDLA chains are prone to adopt more compact conformation during the morphological transformation from LCMs to SLMs. Meanwhile, the PDLA microphase spontaneously evolves from bicontinuous-like structure to a quasi-1D P-twisted ribbon topology in the PS matrix. Notably, PS chains are highly stretched due to overcrowding at the PS/PDLA interface^42^, thus providing the anisotropic driving force for the quasi-1D growth of PDLA lamellar^43^. The P-twisted topology can be rationalized on the basis of tilted chiral lipid bilayers (TCLB) theory which suggests that a compensation between incompatibility and specific interaction of chiral entities triggers the formation of microphase-separated twisted ribbons with helical sense^44–47^. Similarly, the presented PDLA twisted ribbons within SLMs are also proposed to result from chiral transfer of helical PDLA chains which drives right-handed twisting and bending of quasi-1D PDLA ribbons. Such P-twisting triggers orientation stretching of PS chains and thus induces the accumulation of internal stress within the PS matrix of SLMs. We speculated that various complex stretching modes for PS chains could occur during the deformation of LCM into SLM, and the detailed situations for directionally stretching of PS chains (driving force for the chiral rotation of ends of SLM) might be different even though the distinctions could not be clearly observed in experiments (see the schematic descriptions in Supplementary Fig. 19). Reasonably, the accumulated internal stress further relaxes by preferentially twisting the whole SLMs in opposite handedness, which is proposed to be the driving force for the observed symmetric breaking during the closure of SLMs into toroids. Therefore, the above-mentioned closing mode A-L90°&B-L90° is enhanced while A-R90°&B-R90° mode is hindered, thus inducing the majority of M-twisted handedness for PS-*b*-PDLA Moebius strips. Yet, due to the complexity of internal nanostructures of the present micrometer-sized assemblies and unavoidable structural defects within them, the driving force originated from relaxation of internal stress within PS phase is not strong enough to dominate all the closing process of SLMs through the formation of M-twisted Moebius strips. Thus, PS-*b*-PDLA toroidal micelles without twists along the ring and a small quantity of P-twisted Moebius strips coexist with M-twisted Moebius strips.

### Hierarchical chiral transfer and helix–helix transition

Notably, four levels of chirality in different length scale could be found in the presented self-assembly of PS-*b*-PDLA on the basis of a general concept in biomacromolecules^48^. The first level is configurational chirality, which is related to the chiral center of carbon atom covalently connected with different chemical groups. The second level of chirality is attributed to right-handed conformation of PDLA blocks. The third level is the so-called phase chirality associated with the P-twisted handedness of PDLA ribbons. The 180° twist along the ring of Moebius strips belongs to the highest (the fourth) level of chirality (assembly chirality). Herein, the handedness of phase chirality (third-level) agrees well with the conformation chirality (second-level) and also the configurational right-handed chiral center (first-level) while the handedness of generated PS-*b*-PDLA Moebius strips is mainly M-twisted, implying an intriguing helix–helix transition occurs during the chiral transfer from microphase to assemblies (Supplementary Fig. 20). In the present case, the first-level chirality may only play roles in inducing the helical conformation of PDLA blocks, and the phase chirality may result from intermolecular packing scheme, i.e., the conformation chirality. As to the highest level chirality (e.g., the twisted topology along the ring of Moebius strip), the helix–helix transition occurs. We have revealed that the assembly chirality originates from preferential twisting of SLM during its closure and the intrinsic driving force is related to relaxation of internal stress induced by orientated stretching of PS blocks along the twisted PS/PDLA interface. Interphase packing scheme of twisted PDLA ribbons is highly disturbed by surrounded PS layer, which implies that the chiral transfer from the third to the forth level in our case occurs through an indirect way which has not been reported to date. Therefore, this study provides clues for revealing the ambiguity existing in systems where different amounts of left- and right-handed assemblies formed from molecules having one specific chiral handle.

## Discussion

In summary, we have demonstrated that BCPs Moebius strips can be prepared by fast self-assembly of chiral diblock copolymers (e.g., PS-*b*-PDLA). The Moebius strips are formed by spontaneous morphological transformation from LCMs to spindle-like micelles (SLMs) then to toroids with a 180° twist along the ring. The transformation of LCMs to SLMs is driven by crystallization of PDLA accompanied with a significant conformation change of PDLA chains from loosely staggered conformation to compact gauche conformation. The 180° twist along the PS-*b*-PDLA toroidal ring is found to result from closing process of SLMs and possess mainly left-handedness. Therefore, an intriguing helix–helix transition occurs during the chiral transfer from microphase to assemblies. Moreover, after selective removal of the PLA microdomains, Moebius strips with mesoporous chiral channels are derived. The chiral mesoporous Moebius strips are promising in chiral recognition and separation, asymmetric catalysis and chromatography of proteins.

## Methods

### Sample preparation

The BCPs Moebius strips were prepared through a fast self-assembly of chiral block copolymer PS-*b*-PDLA in THF/water mixed solvents. Typically, BCPs were completely dissolved in THF with initial polymer concentration of 0.10 wt%. Afterward, water was quickly added to the solution (1.0% (v/v) per second) under mild stirring until reaching the desired water content. Final concentration of the BCPs toroidal micelles was calculated to be 0.077 wt%. The initial colorless solution became milky (e.g., formation of LCMs with hundreds of nanometers), and formed macroscopic aggregates eventually (e.g., physical aggregation of individual micrometer-sized toroids). The suspension was treated by ultrasonication (80 kW for 30 s) to disperse the assemblies. The formation of BCPs toroidal micelles was monitored at varied water content (13, 18, and 23%) after adding water for 5 and 10 min during the self-assembly. The suspension was quickly transferred into excess water (over 10 folds’ volume) to freeze the microstructures of the polymer assemblies before characterizations.

For the BCP toroidal micelles observation by fluorescence microscope, Nile red (~0.001 wt%) was added to the BCPs solution before water addition and observed directly after the formation of toroidal micelles without being frozen by excess water.

PS-*b*-PDLA assemblies were placed in a polypropylene vial and 0.5 M NaOH aqueous solution (methanol/water = 4/6 (v/v)) was poured over the sample. The vial was sealed with sealing tape for 3 days to ensure complete hydrolysis.

### Electron microscopy

Morphologies of the aggregates were observed by TEM (FEI TecnaiG^2^ 20) at an accelerating voltage of 200 kV and TEM (HITACHI HT7700) at 120 kV. 3D TEM (Talos F200X) was operated at an accelerating voltage of 200 kV. SEM (Sirion 200) and SEM (SU8010) operated at an accelerating voltage of 10 kV and 3.0 kV were used to observe the morphology. After an aqueous dispersion was treated by ultrasonication (80 kW for 2 min), the dispersion was dropped on a carbon-coated copper grid. After drying at 30 °C for 1 h, the EM observation was performed.

### General techniques

DLS measurements were performed at a scattering angle of 90° on LLS spectrometer (ALV/CGS-5022) equipped with an ALV-High QE APD detector and an ALV-5000 digital correlator using a He-Ne laser (*λ* = 632.8 nm) as the light source. All the measurements were carried out at room temperature, and the samples were diluted prior to measurement.

WAXS experiments were performed on a modified Xeuss system (Xenocs France), which is equipped with an X-ray source of MetalJet-D2, Excillum on the wavelength of 0.134 nm. A Pilatus 1 M detector (DECTRIS, Swiss, 1480 × 1680 pixels with pixel size of 172 μm) was employed to collect 2D SAXS patterns. The wavelength of the X-ray radiation is 0.154 nm. The sample-to-detector distance was set at 218 mm. SAXS experiments were carried out at the synchrotron beamline BL16B1 (SSRF, Shanghai) with a wavelength *λ* = 0.124 nm. The sample-to-detector distance was set at 1890 mm. Each SAXS pattern was recorded with an exposure time of 6 min whereas each WAXS pattern was recorded with an exposure time of 30 min. The data correction and background subtraction were performed after recording. Powder samples were prepared by dropping the concentrated suspensions of assemblies on polyimide membrane and dried under room temperature.

Element content (C, O) of surface composition of assemblies was measured on an XPS (AXIS-ULTRA DLD-600W, Shimadzu, Japan). Samples were prepared by spinning the concentrated aqueous suspension of assemblies (SSMs, LCMs, SLMs, and toroids of PS-*b*-PDLA) or solution of PDLA_17k_ and PS_21k_ (control experiments) on cleaned silicon wafers.

AFM images were recorded at a tapping mode under ambient condition using a Digital Instrument Multimode Nanoscope IIIA.

All CD spectrums were measured using a commercially available spectropolarimeter (JASCO, J-810). FTIR was performed on the sample using a Bruker Equinox 55 spectrometer. Total reflection mode was taken for the IR measurements.

### Others

The homopolymers of PLLA (*M*_n_ = 6 kg mol^−1^, *M*_w_/*M*_n_ = 1.16), PDLA (*M*_n_ = 6.2 kg mol^−1^, *M*_w_/*M*_n_ = 1.15), chiral block copolymers, i.e., PS_202_-*b*-PDLA_97_ (the subscripts refer to the repeat units of the block; *M*_n_ = 35 kg mol^−1^, *M*_w_/*M*_n_ = 1.12), PS_202_-*b*-PDLA_69_ (*M*_n_ = 31 kg mol^−1^, *M*_w_/*M*_n_ = 1.12), PS_202_-*b*-PDLA_118_ (*M*_n_ = 38 kg mol^−1^, *M*_w_/*M*_n_ = 1.14), and PS_202_-*b*-PLLA_69_ (*M*_n_ = 31 kg mol^−1^, *M*_w_/*M*_n_ = 1.10) were purchased from Polymer Source Inc. (Canada). Analytical grade THF was refluxed with sodium and distilled before use. All the other regents are of analytical grade and used as received.

## Supplementary information

Supplementary Information

Description of Additional Supplementary Files

Supplementary Movie 1

Supplementary Movie 2

Source Data

## Data Availability

All relevant data are available from the authors. The source data underlying Figs. 1–7 and Supplementary Figs. 1e and h, 2, 4a–c, 4g, 7f, 8, 16c–e, 18a and b, and 20 are provided as a Source Data file. Supplementary Information is available in the online version of the paper.

## References

[1] Starostin EL, Van der Heijden GHM (2007). The shape of a moebius strip. Nat. Mater..

[2] Baumann A (2018). Motorizing fibres with geometric zero-energy modes. Nat. Mater..

[3] Emmer M (1980). Visual art and mathematics: the moebius band. Leonardo.

[4] Ballon DJ, Voss HU (2008). Classical möbius-ring resonators exhibit Fermion-Boson rotational symmetry. Phys. Rev. Lett..

[5] Kreismann J, Hentschel M (2018). The optical moebius strip cavity: tailoring geometric phases and far fields. Eur. Phys. Lett..

[6] Chang C-W (2010). Optical moebius symmetry in metamaterials. Phys. Rev. Lett..

[7] Wang EJ (2015). Aggregation-induced-emission-active macrocycle exhibiting analogous triply and singly twisted moebius topologies. Chem. Eur. J..

[8] Bauer T (2015). Observation of optical polarization moebius strips. Science.

[9] Tanda S (2002). Crystal topology: a moebius strip of single crystals—a crystalline ribbon of niobium and selenium can be coaxed into a novel topology. Nature.

[10] Mai Y, Eisenberg A (2012). Self-assembly of block copolymers. Chem. Soc. Rev..

[11] Ho R-M, Chiang Y-W, Lin S-C, Chen C-K (2011). Helical architectures from self-assembly of chiral polymers and block copolymers. Prog. Polym. Sci..

[12] Qiu H (2016). Uniform patchy and hollow rectangular platelet micelles from crystallisable polymer blends. Science.

[13] Tritschler U, Pearce S, Gwyther J, Whittell GR, Manners I (2017). 50th anniversary perspective: functional nanoparticles from the solution self-assembly of block copolymers. Macromolecules.

[14] Yu H, Jiang W (2009). Effect of shear flow on the formation of ring-shaped ABA amphiphilic triblock copolymer micelles. Macromolecules.

[15] Pochan DJ (2004). Toroidal triblock copolymer assemblies. Science.

[16] Wang Z, Jiang W (2010). Temperature-induced reversible transformation between toroidal and cylindrical assemblies under shear flow. Soft Matter.

[17] Yang C (2017). Toroid formation through a supramolecular “cyclization reaction” of rodlike micelles. Angew. Chem. Int. Ed..

[18] Qiu HB (2019). Uniform toroidal micelles via the solution self-assembly of block copolymer-homopolymer blends using a “frustrated crystallization” approach. Macromolecules.

[19] Cai J (2018). Hierarchical self-assembly of toroidal micelles into multidimensional nanoporous superstructures. ACS Macro Lett..

[20] Golan R, Pietrasanta LI, Hsieh W, Hansma HG (1999). DNA toroids: stages in condensation. Biochemistry.

[21] Conwell CC, Vilfan ID, Hud NV (2003). Controlling the size of nanoscale toroidal DNA condensates with static curvature and ionic strength. Proc. Natl Acad. Sci. USA.

[22] Gao G (2015). Chirality-assisted ring-like aggregation of Aβ (1–40) at liquid-solid interfaces: a stereoselective two-step assembly process. Angew. Chem. Int. Ed..

[23] Huang H, Chung B, Jung J, Park H-W, Chang T (2009). Toroidal micelles of uniform size from diblock copolymers. Angew. Chem. Int. Ed..

[24] Presa-Soto D, Carriedo GA, de la Campa R, Presa Soto A (2016). Formation and reversible morphological transition of bicontinuous nanospheres and toroidal micelles by the self-assembly of a crystalline-*b*-coil diblock copolymer. Angew. Chem. Int. Ed..

[25] Sandra A, Félix F, Emilio Q, Ricardo R (2014). Nanospheres, nanotubes, toroids, and gels with controlled macroscopic chirality. Angew. Chem. Int. Ed..

[26] Pashuck ET, Stupp SI (2010). Direct observation of morphological tranformation from twisted ribbons into helical ribbons. J. Am. Chem. Soc..

[27] Zhang L, Wang TY, Shen ZC, Liu MH (2016). Chiral nanoarchitectonics: towards the design, self-assembly, and function of nanoscale chiral twists and helices. Adv. Mater..

[28] Hifsudheen M, Mishra RK, Vedhanarayanan B, Praveen VK, Ajayaghosh A (2017). The helix to super-helix transition in the self-assembly of π-systems: superseding of molecular chirality at hierarchical level. Angew. Chem. Int. Ed..

[29] Deng M, Zhang L, Jiang YQ, Liu MH (2016). Role of achiral nucleobases in multicomponent chiral self-assembly: purine-triggered helix and chirality transfer. Angew. Chem. Int. Ed..

[30] Li CY (2001). Left or right, it is a matter of one methylene unit. J. Am. Chem. Soc..

[31] Chen C-K, Lin S-C, Ho R-M, Chiang Y-W, Lotz B (2010). Kinetically controlled self-assembled superstructures from semi-crystalline chiral block copolymers. Macromolecules.

[32] Ku KH (2016). Particles with tunable porosity and morphology by controlling interfacial instability in block copolymer emulsions. ACS Nano.

[33] Wang X (2007). Growth and crystallization of metal-containing block copolymer nanotubes in a selective solvent. Adv. Mater..

[34] Wang X (2007). Cylindrical block copolymer micelles and co-micelles of controlled length and architecture. Science.

[35] Jia L (2018). Creating biomorphic barbed and branched mesostructures in solution through block copolymer crystallization. Angew. Chem. Int. Ed..

[36] Ho R-M (2009). Block copolymers with a twist. J. Am. Chem. Soc..

[37] Shen Z, Jiang Y, Wang R, Liu M (2015). Symmetry breaking in the supramolecular gels of an achiral gelator exclusively driven by π–π stacking. J. Am. Chem. Soc..

[38] Ho R-M (2012). Transfer of chirality from molecule to phase in self-assembled chiral block copolymers. J. Am. Chem. Soc..

[39] Liu H (2018). Conformation-directed micelle-to-vesicle transition of cholesterol-decorated polypeptide triggered by oxidation. J. Am. Chem. Soc..

[40] Zhang J (2005). Crystal modifications and thermal behavior of poly(L-lactic acid) revealed by infrared spectroscopy. Macromolecules.

[41] Tanabe Y, Shimomura M (1990). A2 mode vibration in infrared absorption spectrum of trigonal poly(oxymethylene). Macromolecules.

[42] Zheng JX (2006). Onsets of tethered chain overcrowding and highly stretched brush regime via crystalline-amorphous diblock copolymers. Macromolecules.

[43] Shu RF, Zha LY, Eman AA, Hu WB (2015). Fibril crystal growth in diblock copolymer solutions studied by dynamic monte carlo simulations. J. Phys. Chem. B.

[44] Nakashima N, Asakuma S, Kunitake T (1985). Optical microscopic study of helical superstructures of chiral bilayer membranes. J. Am. Chem. Soc..

[45] Fuhrhop J-H, Schnieder P, Boekema E, Helfrich W (1988). Lipid bilayer fibers from diastereomeric and enantiomeric N-octylaldonamides. J. Am. Chem. Soc..

[46] Ou-Yang ZC, Liu JX (1990). Helical structures of tilted chiral lipid bilayers viewed as cholesteric liquid crystals. Phys. Rev. Lett..

[47] Ou-Yang ZC, Liu JX (1991). Theory of helical structures of tilted chiral lipid bilayers. Phys. Rev. A.

[48] Wang J (2010). Helical crystal assemblies in nonracemic chiral liquid crystalline polymers: where chemistry and physics meet. Ind. Eng. Chem. Res..

