# Supplementary material for: Moebius strips of chiral block copolymers
Source: Nat Commun. 2019 Sep 9;10:4090. doi: 10.1038/s41467-019-11991-3 (PMC6733789; doi:10.1038/s41467-019-11991-3)
Supplement: Supplementary file 1 — Supplementary Information [file 41467_2019_11991_MOESM1_ESM.pdf]

Supplementary Information

Moebius Strips of Chiral Block Copolymers

Geng et al.

Supplementary Figures:

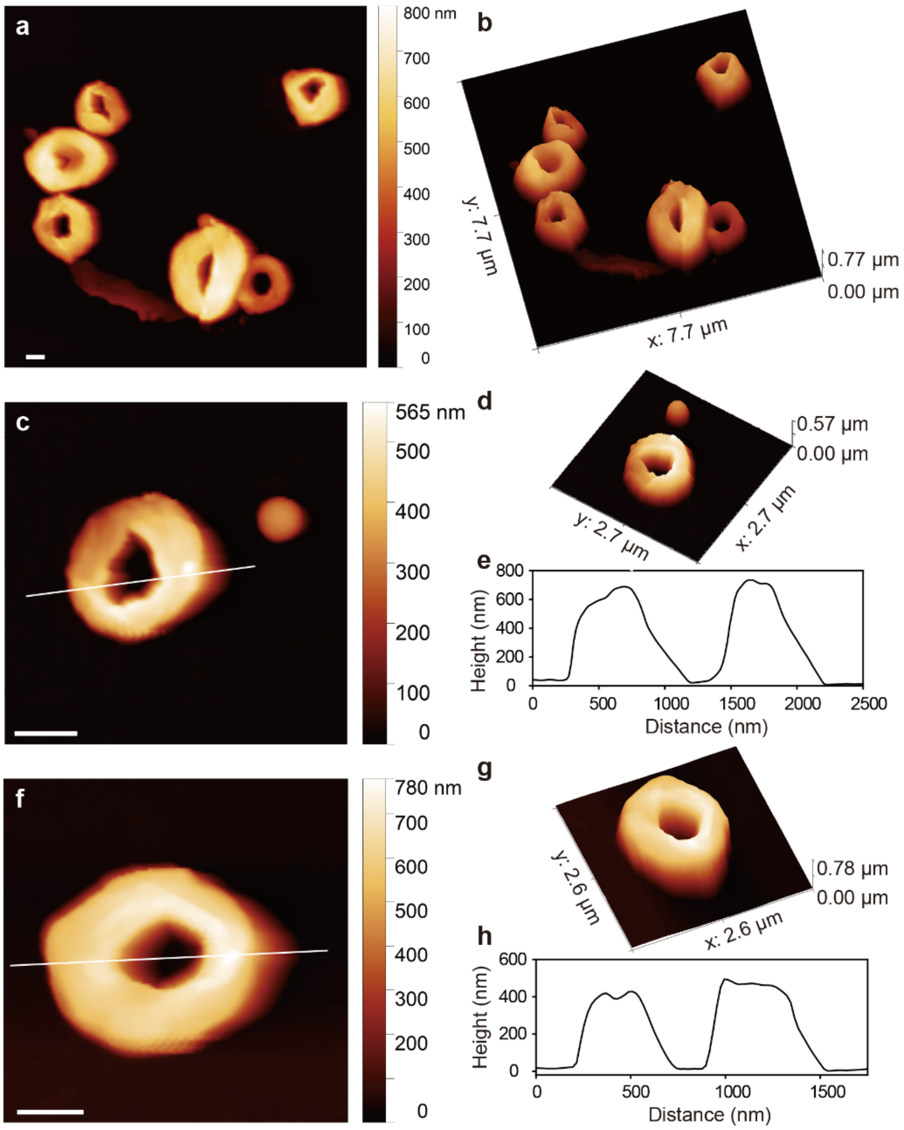

Supplementary Figure 1. Atomic force microscopy (AFM) characterization for toroidal topology of PS-*b*-PDLA assemblies. **a**, AFM image of toroidal assemblies formed from PS-*b*-PDLA. **b**, 3D view for the AFM image in **a**. **c** and **f**, AFM analysis of two isolated toroids. **d** and **g**, 3D views for the AFM images in **c** and **f**, respectively. **e** and **h**, height profiles for the AFM images shown in **c** and **f**. Scale bars: 500 nm.

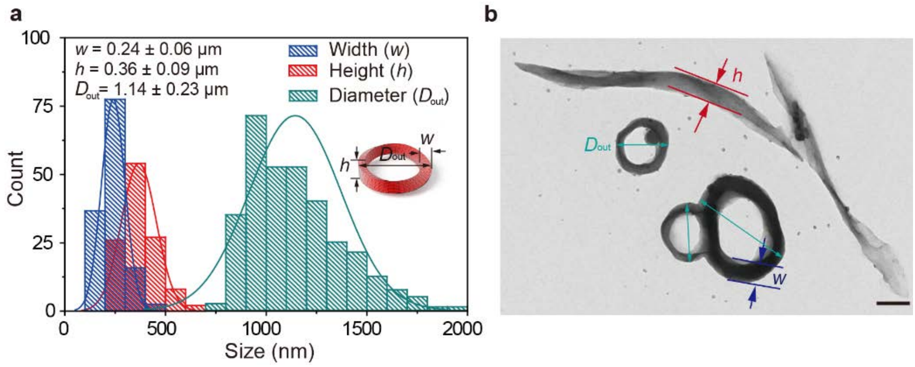

Supplementary Figure 2. Statistical results of geometric parameters of toroids. **a** Statistic histograms of the average outside diameter (D_{out}), ring width (w), and height (h) of the Toroids. **b** A detailed example for the calculations of D_{out}, w, and h. Inseted cartoon image in (**a**) schematically showing the geometric parameters of a toroid. Notably, h is equivalante with width of spindle like micelle (SLM) and thus the average value are obtained by calculating the width of SLMs for replacement. Each measurement is operated on all the aggregates in 5 different TEM images and the statistical amount for D_{out}, w, and h are larger than 300.

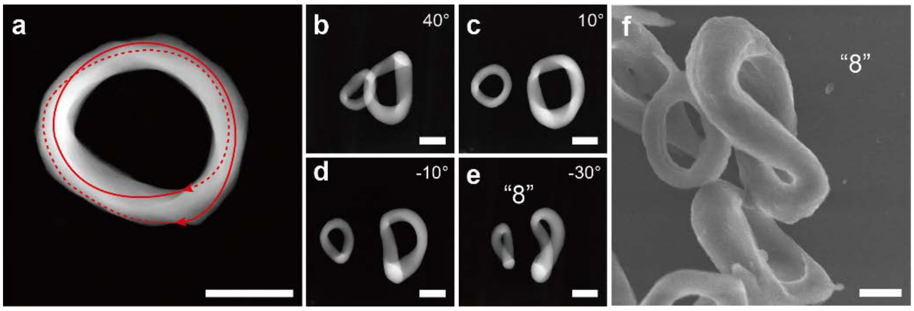

Supplementary Figure 3. Electron microscopy of the Moebius strips to revealing their twisted topology along the ring. **a**, Two successive cycled traces along the wider face on the Moebius strip ring; **b** – **e**, serials of STEM images of Moebius strips under different view angles, and a figure-of-eight shaped structure from the side view can be observed; **f**, SEM image of a figure-of-eight shaped structure from the side view. Scale bars: 500 nm.

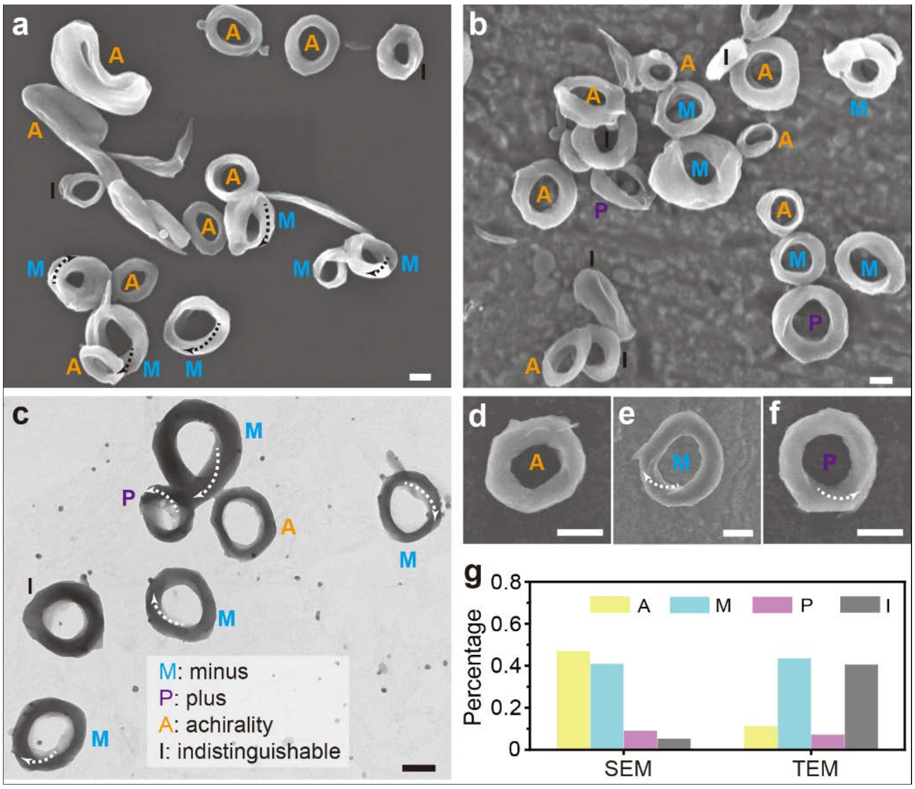

Supplementary Figure 4. Investigation for statistical percentages of twisted topology along the ring of toroidal assemblies. **a** and **b**, Representative SEM image for PS-*b*-PDLA Moebius strips with marks for twisted structure. **c**, Representative TEM image for clearly showing the twisted structure. **d** to **f**, Enlarged SEM images for toroidal micelle without twists, M-twisted Moebius strip, and P-twisted Moebius strip, respectively. **g**, Histogram for the counted results of the ratios of twisted structures for PS-*b*-PDLA Moebius strips from SEM and TEM images. Notably, more than 200 toroids were counted to obtain these statistical results. Scale bars: 500 nm.

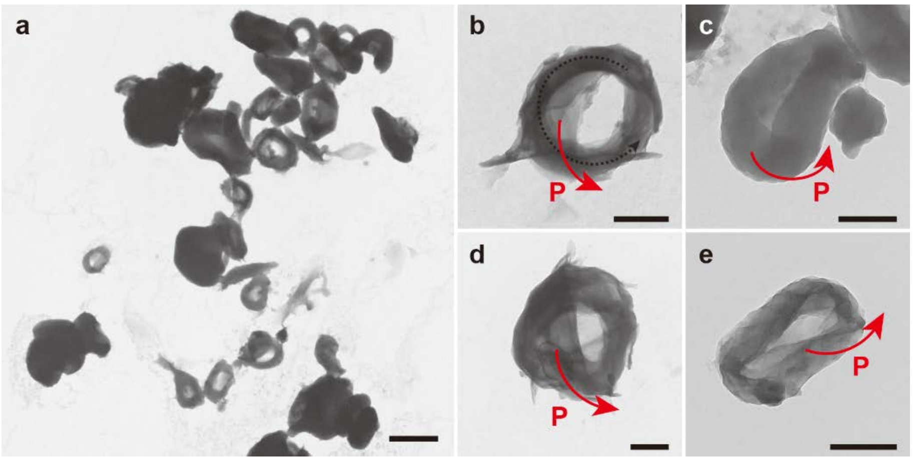

Supplementary Figure 5. Toroidal assemblies of PS-*b*-PLLA. **a**, Representative TEM image of PS-*b*-PLLA toroidal assemblies. **b** – **e**, representative TEM images with high magnification of P-twist PS-*b*-PLLA Moebius strips. Scale bars: 2 μm in **a**; 500 nm in **b** – **e**.

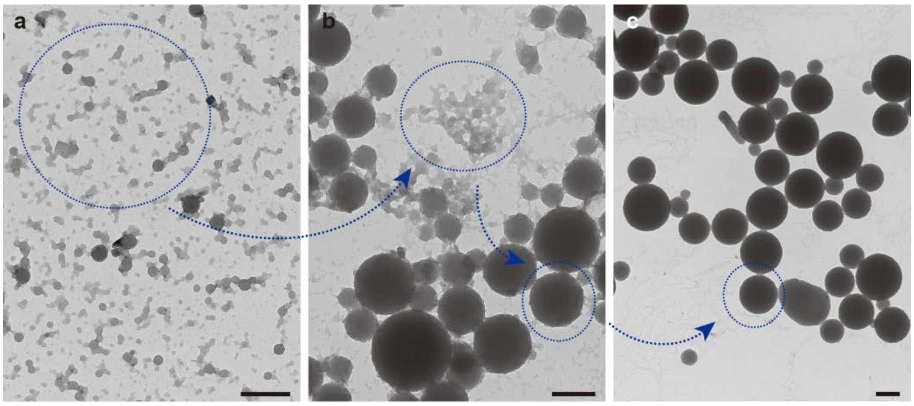

Supplementary Figure 6. Structural evolution of PS-b-PDLA assemblies into Large compound micelles (LCMs). **a**, Representative TEM images for SSMs. **b**, aggregates of SSMs and LCMs with rough surface. **c**, LCMs with smooth surface. These intermediate assemblies were obtained by freezing the morphologies of aggregates when the water addition content reached 13.8% (v/v), 15.3%, and 18.0%, respectively. Possible formation process of LCMs was suggested by a series of sequenced dashed rings and curved arrows. Scale bars: 500 nm.

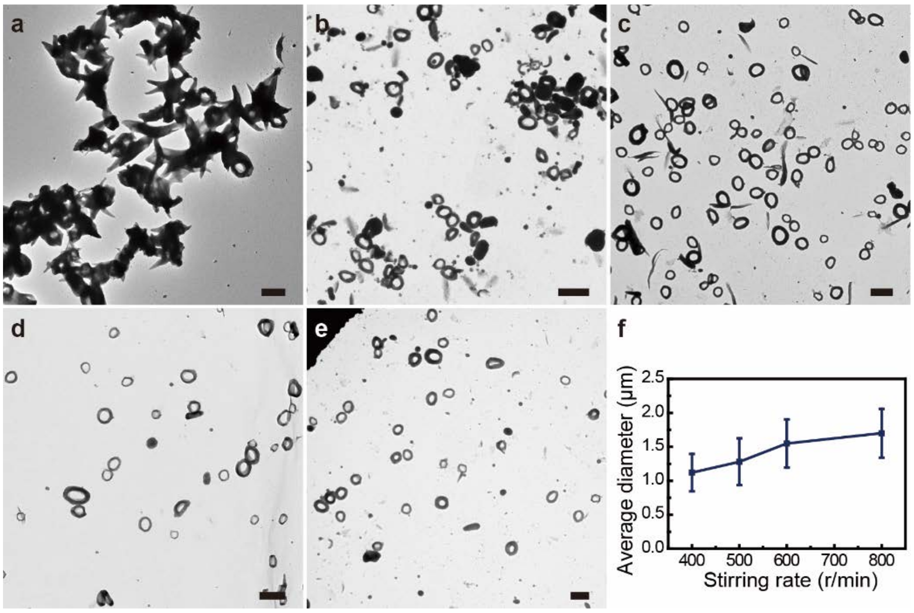

Supplementary Figure 7. TEM images of the assemblies obtained by a fast self-assembly of PS-*b*-PDLA under different stirring conditions: **a**, stirring at 500 rpm during the addition of water while stirring stops upon completion of water addition; **b**, the stirring rate was 400 rpm; **c**, 500 rpm; **d**, 600 rpm; **e**, 800 rpm; **f**, statistic average diameters of BCP Moebius strips against the stirring rate. Error bars represent the s.d. Scale bars: 2 μm.

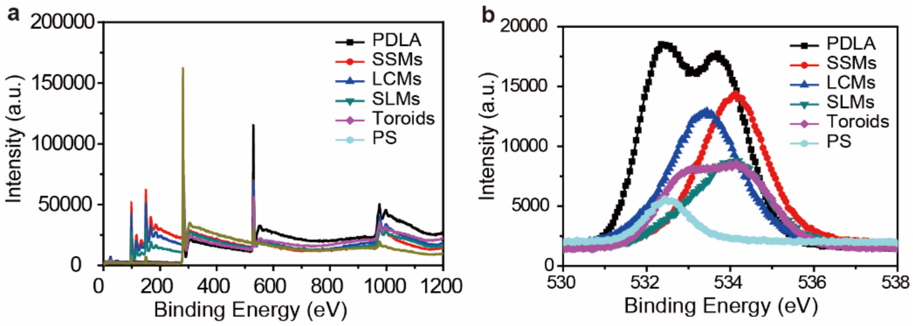

Supplementary Figure 8. X-ray photoelectron spectroscopy for PS and PDLA film and intermediate assemblies obtained during the formation of PS-*b*-PDLA toroids. **a**. Full spectra and **b**. XPS O 1s spectra.

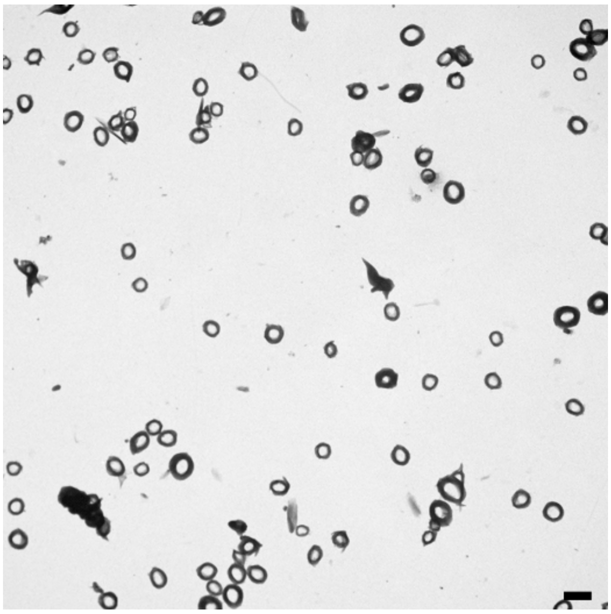

Supplementary Figure 9. TEM image of the BCP* Moebius strips after being stored in THF/water mixture (77/23, v/v) for 6 months. Scale bar: 2 μm.

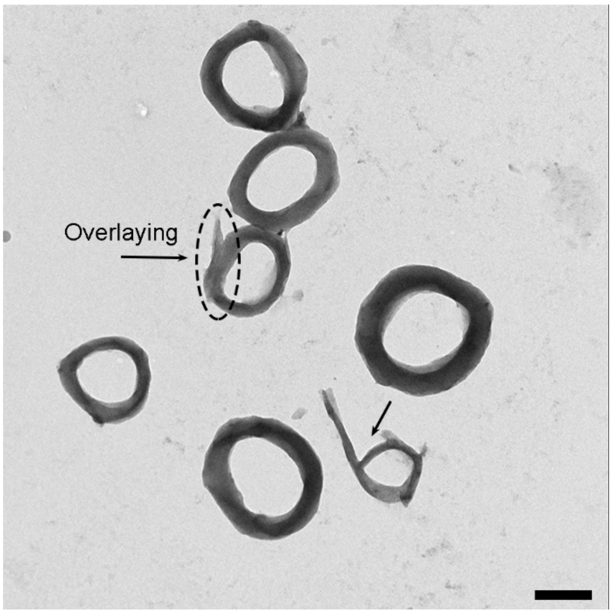

Supplementary Figure 10. TEM image of the structure with a tail at the junction during the end-to-end closure of spindle-like micelle. Scale bar: 500 nm.

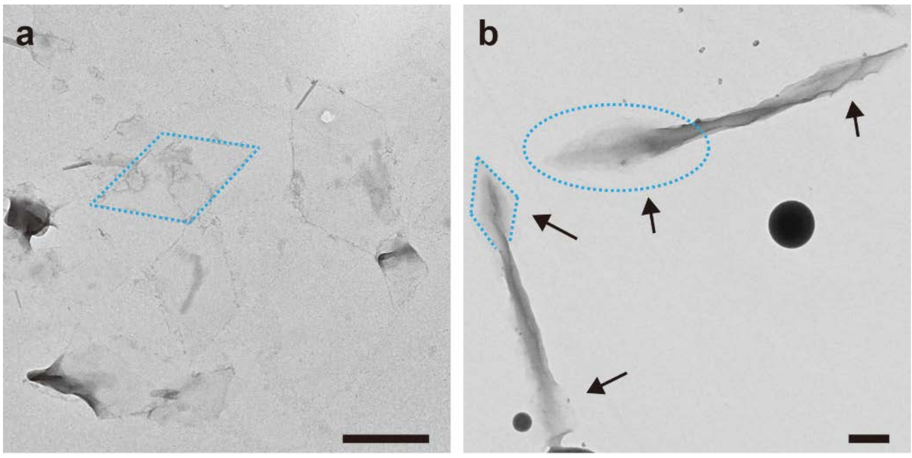

Supplementary Figure 11. TEM images of **a**, a lozenge lamellar crystal by the self-assembly of PS-*b*-PDLA in the case of slow water addition rate (0.2 % (v/v) per min); **b**, the spindle-like micelles with two lozenge crystal-like tails indicated by the black arrows. Scale bars: 500 nm.

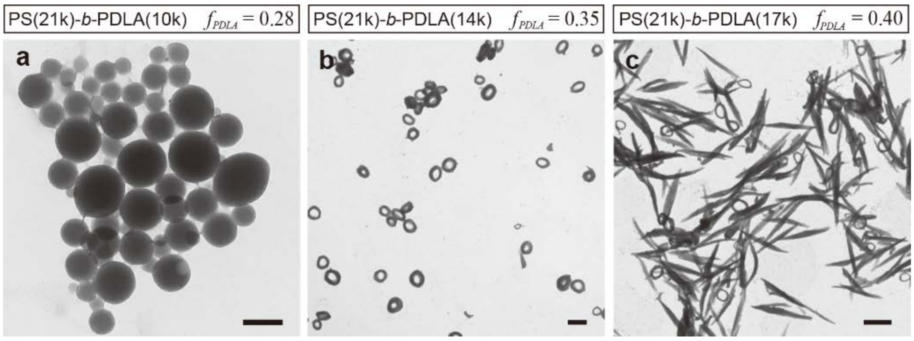

Supplementary Figure 12. Representative TEM images for the self-assembled morphologies of PS-*b*-PDLA with different PDLA volume fraction (f_{PDLA}) in THF/water mixture (77/23, v/v). Scale bars: 500 nm in **a**; 2 μm in **b** and **c**.

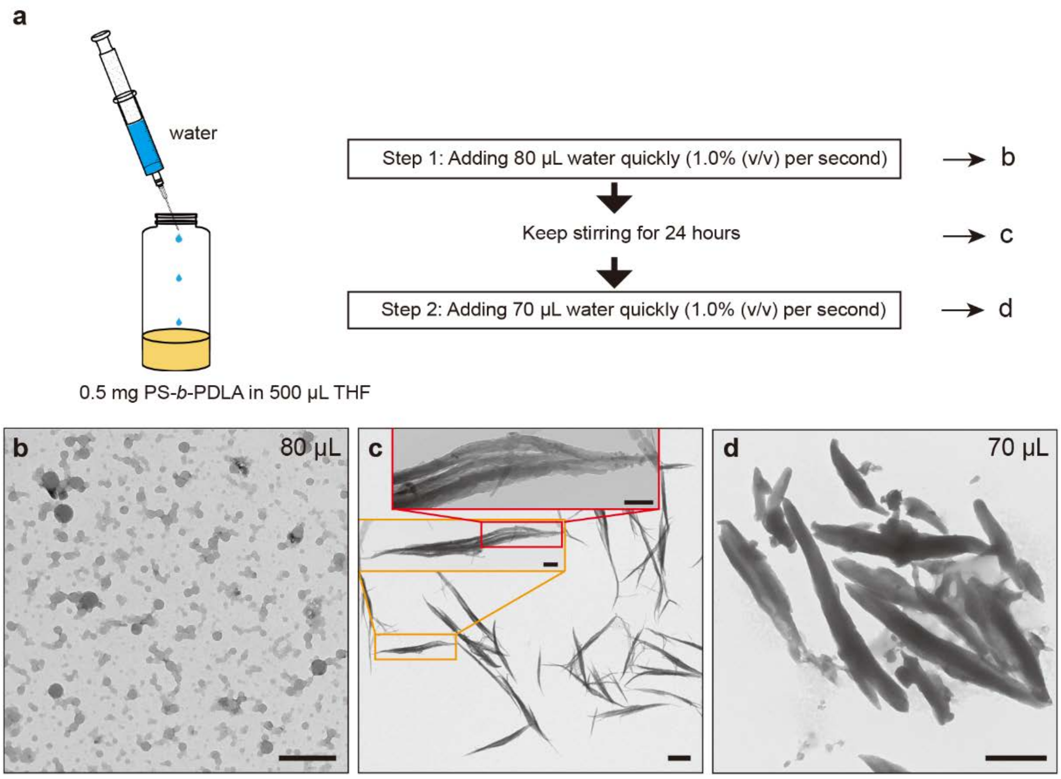

Supplementary Figure 13. A designed two-step assembly process of PS-*b*-PDLA as control experiments. **a.** Schematic cartoon description of a designed two-step assembly process. **b–d**, representative TEM images for morphologies of assemblies obtained when the water addition contents reached 13.8% (v/v) (**b**), after stirring for 24 h (**c**), and after water content reached 23.1% (the predetermined final water content) (**d**). Scale bars: 500 nm in **b** and insets; 2 μm in **c** and **d**.

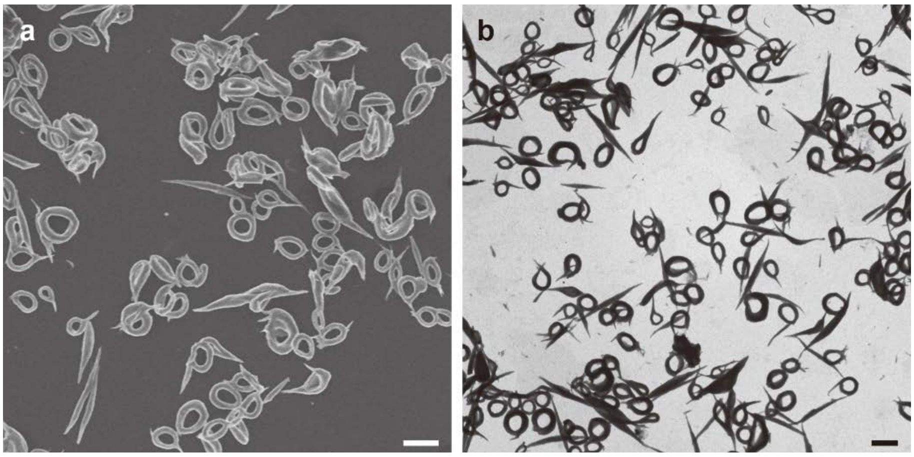

Supplementary Figure 14. EM images of intermediate assemblies onbtained during the morphological evolution from SLMs to toroids. **a** and **b**, Representative SEM and TEM images of captured intermediate state for the closing of SLMs into toroids. Scale bars: 2 μm.

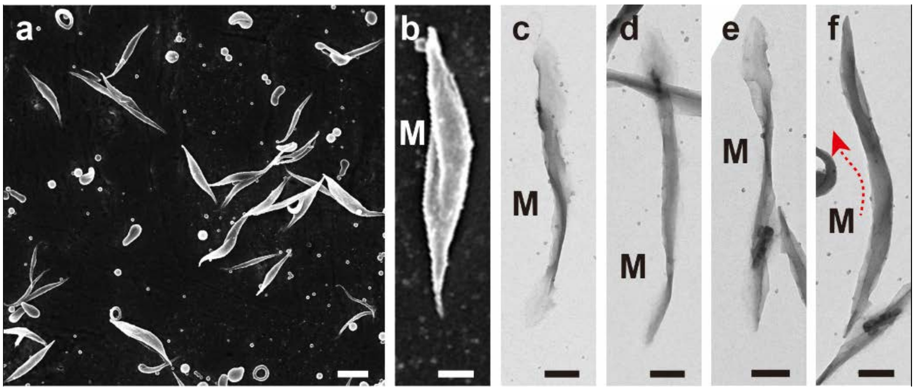

Supplementary Figure 15. Representative EM images of SLMs with 180° M-twist. **a** and **b**, SEM images of the SLMs with a left-handed twist (M-twisted) along the long axle; **c - f**, magnified TEM images for M-twisted SLMs. Scale bars: 2 μm in **a**; 500 nm in **b – f**.

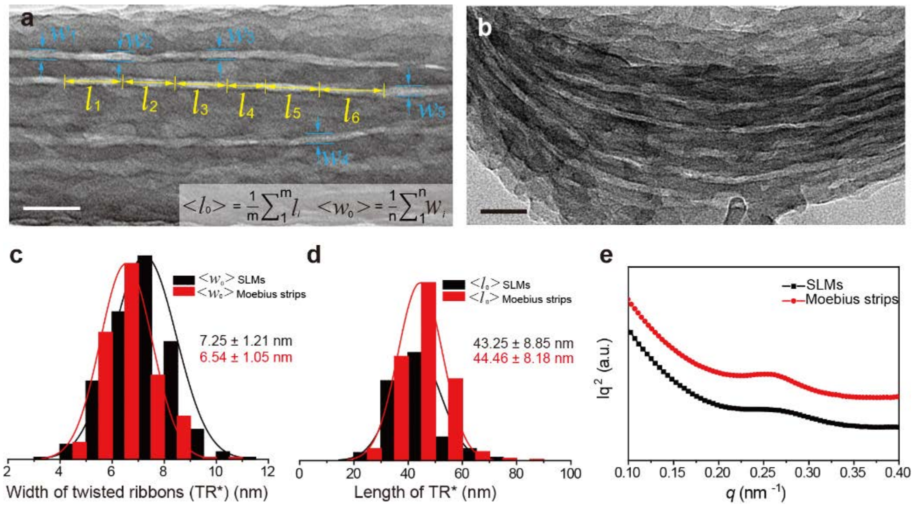

Supplementary Figure 16. Detailed characterization of PDLA nanostructures within the SLM and toroid. **a** and **b**, Representative TEM images with high magnification for PDLA ribbons within SLM and toroidal micelle with schematic descriptions of pitch length (l_0) and width (w_0), respectively. **c** and **d**, Counted result of average l_0 and w_0 for PDLA ribbons with SLMs and toroidal micelles, respectively. **e**, SAXS spectra for SLMs and toroidal micelles. Scale bars: 50 nm.

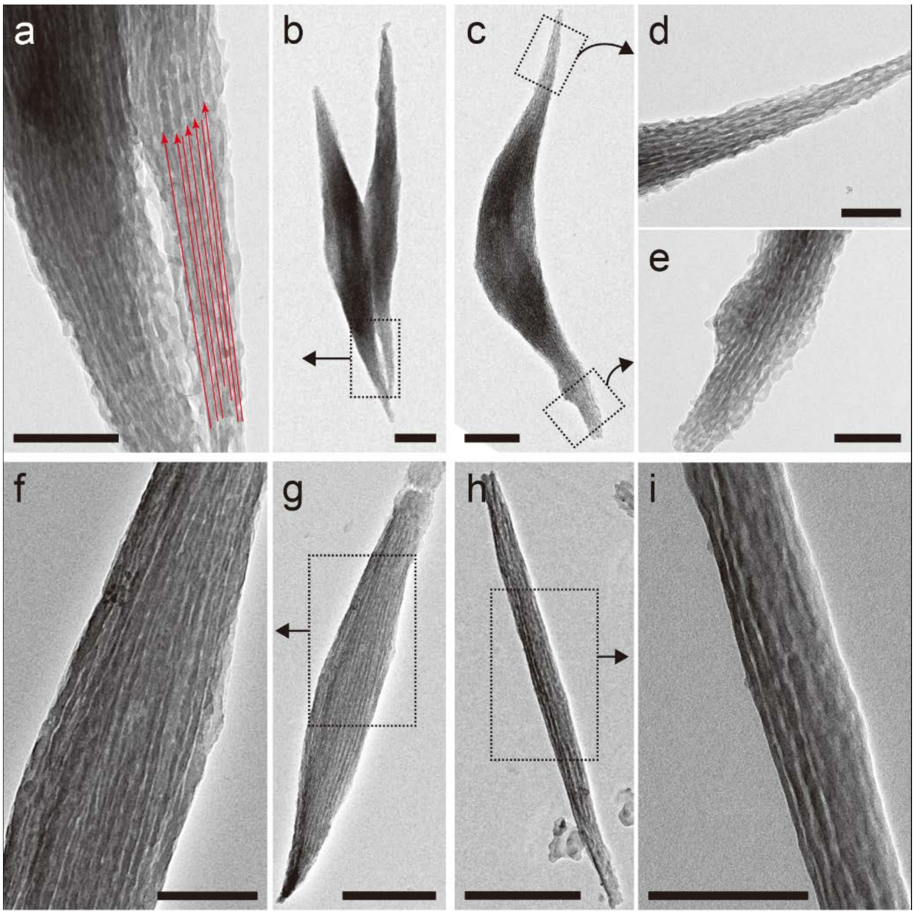

Supplementary Figure 17. Representative TEM images for SLMs after selectively etching the PDLA microphase. Red arrows in **a** indicate the quasi-1D right-handed twisted PDLA ribbons stacked within PS matrix in a roughly parallel manner. Scale bars: 200 nm in **a**, **d** – **f**, and **i**; 500 nm in **b**, **c**, **g**, and **h**.

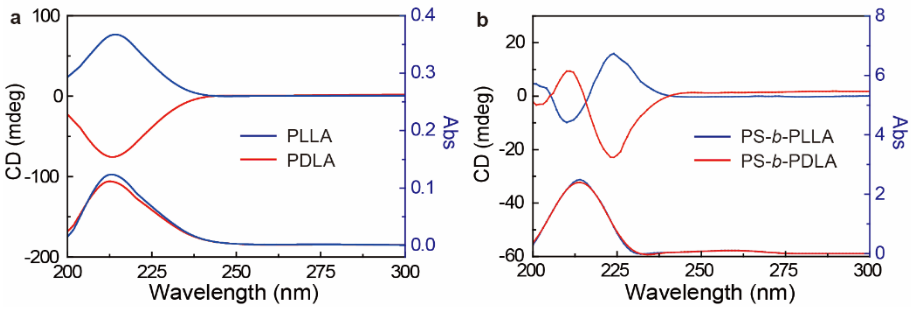

Supplementary Figure 18. CD and corresponding UV-vis spectra of **a**, polylactide homopolymers, and **b**, PS-*b*-PDLA and PS-*b*-PLLA in dilute THF solution.

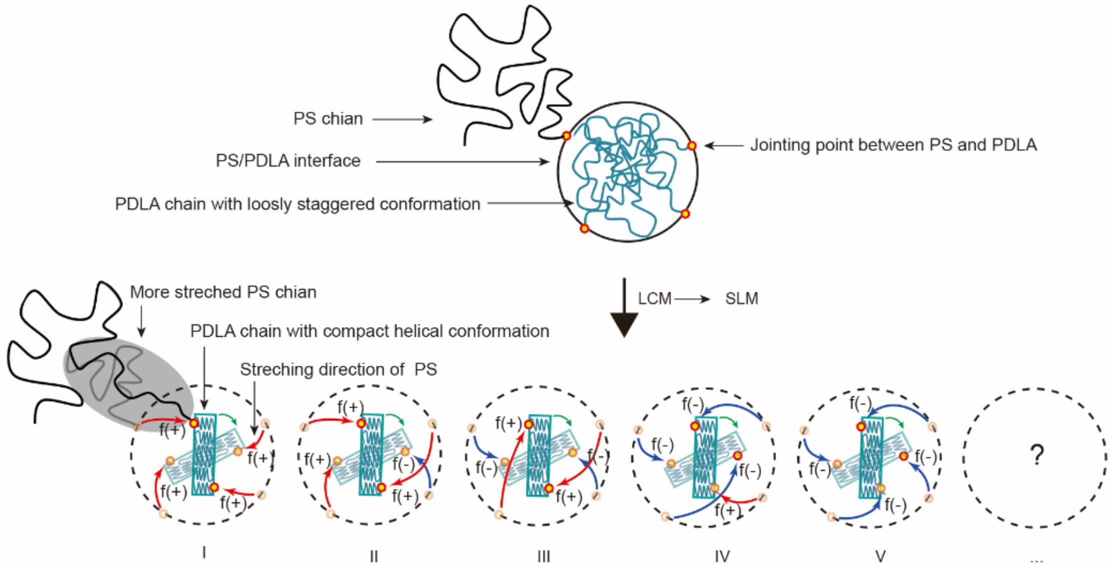

Supplementary Figure 19. Schematic description for orientation stretching of PS chains during the spontaneous evolution of PDLA microphase from bicontinuous-like structure to a quasi-1D P-twisted ribbon topology. I and II, the stretching force in total within the microdomains was along clockwise direction which would be relaxed by opposite rotation of SLM; III, the stretching forces were quits; IV and V, the stretching force in total was along counterclockwise direction which might correspond to the SLMs with P-twisted rotation.

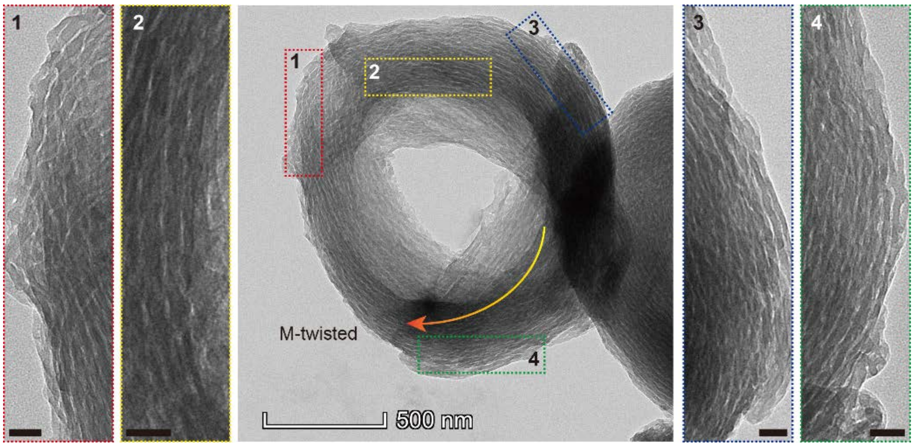

Supplementary Figure 20. Representative TEM images with high magnification for M-twisted PS-*b*-PDLA Moebius strip and P-twisted PDLA ribbons within it. Scale bars in 1, 2, 3 and 4: 50 nm.

Supplementary Table:

Supplementary Table 1. Peak positions and atomic ratio of the XPS C 1s and O 1s for PS and PDLA films (as control results), SSMs, LCMs, SLMs, and toroids of PS-*b*-PDLA.

	Peak Position (eV)	Atomic Ratio (%)					
		PDLA	SSMs	LCMs	SLMs	Toroids	PS
C 1s	283.3	71.9	65.2	75.9	86.3	86.5	97.6
O 1s	531.5	28.1	34.8	24.1	13.7	13.5	2.4
