# Supplementary material for: Moebius strips of chiral block copolymers
Source: Nat Commun. 2019 Sep 9;10:4090. doi: 10.1038/s41467-019-11991-3 (PMC6733789; doi:10.1038/s41467-019-11991-3)
Supplement: Supplementary file 2 — Description of Additional Supplementary Files [file 41467_2019_11991_MOESM2_ESM.pdf]

Description of Additional Supplementary Files

File name: Supplementary Movie 1

Description: STEM tomography video of a typical PS-b-PDLA Moebius strip

File name: Supplementary Movie 2

Description: Reconstructed 3D image of a typical PS-b-PDLA Moebius strip.
